# Fluid and Electrolyte Disturbances in COVID-19 and Their Complications

**DOI:** 10.1155/2021/6667047

**Published:** 2021-04-14

**Authors:** Mohammad Pourfridoni, Seyede Mahsa Abbasnia, Fateme Shafaei, Javad Razaviyan, Reza Heidari-Soureshjani

**Affiliations:** ^1^Student Research Committee, Jiroft University of Medical Sciences, Jiroft, Iran; ^2^Student Research Committee, Department of Clinical Biochemistry, School of Medicine, Shahid Beheshti University of Medical Sciences, Iran; ^3^Students' Scientific Research Center, Tehran University of Medical Sciences, Tehran, Iran

## Abstract

The novel coronavirus disease 2019 (COVID-19) is the cause of an acute respiratory illness which has spread around the world. The virus infects the host by binding to the angiotensin-converting enzyme 2 (ACE2) receptors. Due to the presence of ACE2 receptors in the kidneys and gastrointestinal (GI) tract, kidneys and GI tract damage arising from the virus can be seen in patients and can cause acute conditions such as acute kidney injury (AKI) and digestive problems for the patient. One of the complications of kidneys and GI involvement in COVID-19 is fluid and electrolyte disturbances. The most common ones of these disorders are hyponatremia, hypernatremia, hypokalemia, hypocalcemia, hypochloremia, hypervolemia, and hypovolemia, which if left untreated, cause many problems for patients and even increase mortality. Fluid and electrolyte disturbances are more common in hospitalized and intensive care patients. Children are also at greater risk for fluid and electrolyte disturbances complications. Therefore, clinicians should pay special attention to the fluid and electrolyte status of patients. Changes in fluid and electrolyte levels can be a good indicator of disease progression.

## 1. Introduction

The 2019 novel coronavirus (2019-nCoV), which has caused the spread of a respiratory pandemic, for the first time, was renamed by the Chinese government as the novel coronavirus pneumonia. The World Health Organization (WHO) later named the emerging disease COVID-19 [1]. Since March 6, 2021, over 115 million cases have been reported globally, with more than 2 million deaths [2]. Coronaviruses are a group of nondividing positive-sense RNA viruses that pertain to the class Coronaviridae and are pathogenic in mammals, especially humans [3]. Severe acute respiratory syndrome coronavirus 2 (SARS-CoV-2) is known as the seventh type of coronavirus class of human infection [4]. Six other types of human coronavirus can cause diseases such as SARS-CoV and coronavirus Middle East respiratory syndrome (MERS-CoV) with high mortality [5]. The most common symptoms of COVID-19 include fever, dry cough, fatigue, muscle aches, and other symptoms such as shortness of breath, headache, diarrhea, and indigestion [6–9]. From the onset of symptoms to death from the disease is between 6 and 41 days, and the average is 14 days. This time is shorter than patients under 70, depending on the patient's age and immune status [6]. So far, there is no comprehensive antiviral treatment or antiviral drug for COVID-19 [10]. Disease control includes therapeutic measures to control symptoms, supportive and protective measures, disruption of the disease transmission chain by quarantining patients, and experience-based therapy [11]. The WHO has announced COVID-19 outbreak an emergency state [12].

Previous studies have shown that in COVID-19, in addition to the respiratory system, the nervous system, cardiovascular system, gastrointestinal (GI) tract, and urogenital system are also affected by the disease and its complications. Because the GI tract and kidneys play an essential role in fluid and electrolyte balance in the body, disturbance can lead to an imbalance of fluid and electrolytes. Impaired fluid and electrolyte balance can be dangerous if left unchecked.

## 2. Materials and Methods

This study is aimed at investigating the fluid and electrolyte disturbances in COVID-19 patients and the complications that may occur following these disorders in patients. The literature search for this article has been entirely Internet-based. Web of Science, PubMed, Scopus, and Google Scholar databases were used to collect information. List of terms and phrases, including acute kidney injury (AKI), acute renal injury, renal insufficiencies, renal impairment, renal failures, angiotensin-converting enzyme 2 (ACE2), SARS-CoV-2, COVID-19, 2019-nCoV infection, fluid and electrolyte disturbance, water-electrolyte imbalances, was used for a more focused search. There was no restriction on the date, place, type of study, and inclusion/exclusion criteria for reviewing articles. Studies not written in English were withdrawn.

## 3. Results and Discussion

### 3.1. How the SARS-CoV-2 Affects the Kidneys and GI Tract

The coronavirus family causes infections via respiratory droplets, contact, etc. [13]. The ACE2 receptor is a receptor that the SARS-CoV-2 uses, like the SARS-CoV, to perform its pathogenicity [14, 15]. Coronavirus S protein is known to inhibit virus entry into cells [16–18]. Protein S binds the receptor to the membrane fusion. This protein performs a very crucial function in determining the number of host tropics and transmission capacity. To function, protein S is split into domains S1 and S2, which perform receptor binding and fusion of cell membranes, respectively [18]. SARS-CoV-2 can cause kidney injury by having a direct effect on the kidney tissue, as ACE2 and members of the serine protease family are intensely expressed on podocytes and tubule epithelial cells [19]. Also, virus entry into GI epithelial cells is mediated by the spike protein on the viral coating initiated by the cellular transmembrane serine protease 2 (TMPRSS-2) [20]. Therefore, the involvement of the kidneys and GI tract in COVID-19 is possible.

### 3.2. Kidney Involvement in COVID-19

The kidneys have a wide range of roles, including urine formation, hormone secretion, blood pressure regulation, acid-base balance, and osmolality regulation, so their normal function is essential [21]. Acute renal impairment due to COVID-19 is not unexpected. As mentioned, coronavirus enters the cell by binding to ACE2 receptors [14, 15]. Due to the high level of these receptors in kidney cells, the kidneys are not immune to coronavirus invasion. The possible mechanisms that cause renal impairment in cases with COVID-19 are rooted in fluid imbalance due to fever or decreased fluid intake in patients [22]. Recent reports indicate that renal impairment is more common in patients with COVID-19 [8, 22, 23]. The cytopathic effects of COVID-19 on podocytes and proximal tubular straight cells could cause AKI in sufferers with COVID-19, especially in patients whose virus is found in their blood sample. Podocytes and proximal tubular straight cells play an essential role in normal renal function by filtering urine, absorbing, and secreting. Podocytes are much more sensitive to viral and bacterial invasions and are at greater risk. Therefore, more consideration should be paid to new surveillance of renal function and urinary management of COVID-19 patients with AKI to avert unintended infections [24]. Kidney disorders in COVID-19 occur as a result of complex factors. It is important to note that AKI is correlated with considerable mortality in sufferers with COVID-19 [22]. Because many electrolyte disorders have significant consequences to help identify the pathophysiological mechanisms underlying COVID-19 and patient management, they can provide new therapeutic opportunities [25]. The kidneys' involvement with pathogens can disrupt a wide range of body mechanisms and cause many problems such as fluid and electrolyte imbalances. Therefore, monitoring renal function can prevent severe complications in patients involved with COVID-19 and operates an essential role in reducing mortality. A collection of recent studies shows that the most common renal complication in COVID-19 hospitalized patients is electrolyte disorders, especially hyperkalemia [26].

### 3.3. GI Tract Involvement in COVID-19

Based on retrospective study data, the prevalence of GI symptoms in COVID-19 patients could be between 11.4 and 50% [20]. Although COVID-19 is known to be a respiratory disease, there is ample evidence that it can damage the GI tract and cause GI symptoms. In patients with COVID-19, the SARS-CoV-2 can damage the normal intestinal mucosa and disrupt its normal function, leading to GI symptoms and difficulty absorbing nutrients. SARS-CoV-2 can also impair the digestive system of the normal intestinal flora and lead to various GI symptoms, especially diarrhea [27]. The ileum and colon can become dysfunctional due to SARS-CoV-2 infection, which leads to multiple manifestations of GI in patients. The most common of these symptoms are diarrhea, nausea, vomiting, and abdominal discomfort [28]. Evidence has shown that SARS-CoV-2 infection is associated with changes in the gut microbiota. Clinical studies on COVID-19 have demonstrated that in COVID-19 patients, GI symptoms can precede respiratory symptoms [29]. GI disorders in COVID-19 patients can lead to fluid and electrolyte disturbance [1, 14, 24, 30–32].

### 3.4. Fluid and Electrolyte Disturbance in COVID-19

Impaired renal function leads to fluid and electrolyte disturbances [33]. GI disorders can also lead to fluid and electrolyte imbalances (the most common type is hypokalemia) [34]. Controlling fluid and electrolyte balance involves several processes in which the kidneys and GI tract play an essential role. As a result, damage to them usually disrupts fluids and electrolyte balance [35].

Studies on COVID-19 confirm electrolyte disturbances in patients, including sodium, potassium, chlorine, and calcium imbalances [8, 31]. One of the most common electrolyte disorders is hyponatremia, which occurs with a heightened risk of mortality in hospitalized patients [36]. Some drugs previously used in the United States Food and Drug Administration's (FDA) treatment protocol for patients with COVID-19, such as chloroquine and hydroxychloroquine, can cause electrolyte imbalance [37]. People with COVID-19, who are taking drugs that inhibit the RAS, reduce the production of aldosterone, and this can cause fluid and electrolytes imbalances in the patient. Mineralocorticoid receptor (MR), which has different types, is expressed in various tissues, including the kidneys, GI tract such as the colon, central nervous system (CNS), and heart, and is known as aldosterone receptor. Activation of MR leads to changes in the concentration of ions (such as sodium and potassium). These changes are necessary to maintain the balance of fluid and electrolytes in the body. Still, due to MR's presence in the large intestine [38–40], if the aldosterone pathway is disrupted, the absorption and secretion of ions in the colon are disrupted, and fluid and electrolyte imbalance occurs. A case-control study showed that hyponatremia, hypokalemia, and hypochloremia, which are electrolyte disturbances, were more common in COVID-19 patients than in controls [41]. Hypokalemia, a complication of COVID-19, can exacerbate acute respiratory distress syndrome (ARDS) and increase the risk of heart injuries in patients [25]. Hypernatremia is very common in patients with acute COVID-19. Patients with hypernatremia have been in intensive care for a more extended period and have a higher risk of death. Therefore, the amount of ions, including sodium and potassium, is a significant indicator in COVID-19 patients [42]. Hypocalcemia is also one of the electrolyte disorders in patients with COVID-19, which can be dangerous if not controlled and can even increase the mortality rate [43]. In some people with SARS-CoV-2 infection, the syndrome of inappropriate antidiuretic hormone secretion (SIADH) has been reported [44], leading to disturbances in fluid and electrolytes.

### 3.5. Fluid and Electrolyte Disturbance Complications

Proper electrolyte balance is essential for regulating body functions and sustaining health. Even the slightest deviation from average electrolyte concentrations can cause many problems and sometimes carry death risk. Immediate and decisive treatment is required for fluid and electrolyte disturbances [45]. Fluid and electrolyte disturbances are prevalent in patients admitted to the intensive care unit (ICU). Acute cerebral edema is a disastrous complication for patients with hyponatremia [46]. Rhabdomyolysis, seizures, mood swings, and even coma are some of the difficulties of hyponatremia in patients. Rapid correction of hyponatremia or hypernatremia can lead to the demyelinating osmotic syndrome, so physicians should pay special attention to this issue [46, 47]. Hypokalemia is very destructive if left unchecked and affects the cardiovascular function, neurohormonal activation, and other vital organs [48]. The most common complication of hypocalcemia is increased neuromuscular irritability, which is characterized by muscle spasms, tingling in the limbs, and perioral numbness. Also, hypocalcemia can rarely cause reversible cardiomyopathy [49]. One of the serum electrolytes is chloride; the change in its concentration can increase the risk of acute kidney injury, morbidity, and even mortality [50]. Hypovolemia and hypervolemia can occur in patients with body fluid disturbance [51]. Hypovolemia, if not controlled and treated promptly, leads to ischemic injury of the vital organs and organ failure [52]. Hypervolemia is a life-threatening factor (especially in children), regardless of the severity of the disease [53].

## 4. Conclusions

In COVID-19, the kidneys and GI tract are at risk, and a variety of complications have been reported that are very common [54, 55]. Fluid and electrolyte disturbances are complications of the kidney and GI injuries in COVID-19 patients. Because fluid and electrolyte disturbances can lead to many problems and even death, clinicians should have special supervision over fluid and electrolyte balance in COVID-19 patients, especially in patients under intensive care because the risk of fluid and electrolyte disturbance is higher in them and it can raise mortality rate [42]. Hyponatremia, hypernatremia, hypokalemia, hypocalcemia, hypochloremia, and changes in fluid body volume are the most common fluid and electrolyte disorders in SARS-CoV-2 infection that should be given special attention. If these disorders are observed, definitive and immediate treatment should be started. Since fluid and electrolyte disturbances can be seen in COVID-19, body fluid volume and electrolyte concentrations can be used to measure disease status and disease progression.
